# Protocol for SEC-based isolation and characterization of human plasma-derived extracellular vesicles

**DOI:** 10.1016/j.xpro.2026.104735

**Published:** 2026-07-23

**Authors:** Fanni Annamária Boros, Philipp Arnold, Martin Regensburger, Friederike Zunke

**Affiliations:** 1 Department of Molecular Neurology, University Hospital Erlangen, Friedrich-Alexander-University, Erlangen-Nürnberg (FAU), 91054 Erlangen, Germany; 2 Institute of Functional and Clinical Anatomy, Friedrich-Alexander-University, Erlangen-Nürnberg, 91054 Erlangen, Germany

**Keywords:** Cell isolation, Microscopy, Neuroscience, Protein Biochemistry

## Abstract

Extracellular vesicles (EVs) are gaining increasing attention among studies focusing on biomarker research, elucidating disease mechanisms, and exploring therapeutic approaches. Here, we present a method for size-exclusion chromatography (SEC)-based enrichment of human plasma EVs, and describe steps for assessing the quality of obtained EV samples. For characterizing the EVs, we provide detailed procedures for western blot, transmission electron microscopy, and nanoparticle tracking analysis techniques.

## Highlights

• Procedures for human plasma extracellular vesicle (EV) isolation and characterization
• Steps for SEC column preparation and plasma EV fractionation
• Instructions for EV sample characterization via WB, TEM, and NTA

## Abstract (editor highlights)

Publisher’s note: Undertaking any experimental protocol requires adherence to local institutional guidelines for laboratory safety and ethics.

## Graphical abstract

**Figure:**
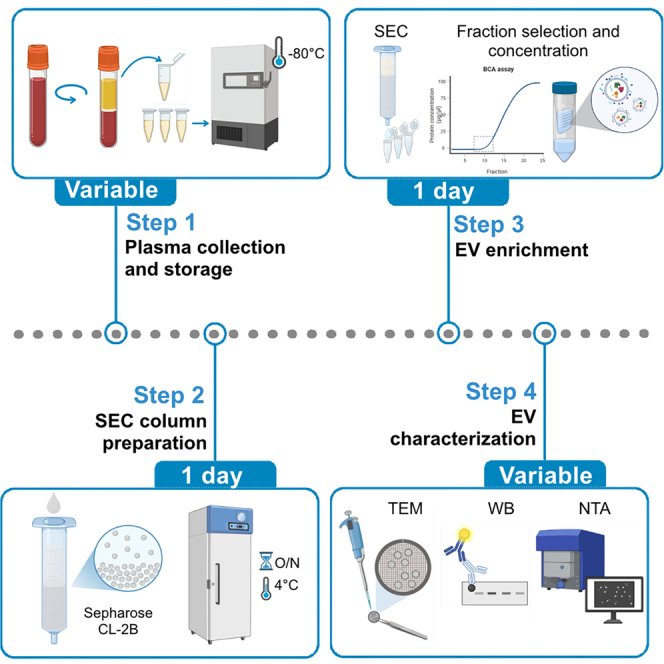

## Before you begin

Extracellular vesicles (EVs), by definition, are cell-released particles, which are bound by a lipid-bilayer and are not able to replicate. The membrane- and the internal, cargo-content of EVs is characteristic of the cells they originate from.^1^^,^^2^^,^^3^ This makes them attractive sources of information on the status of organs hardly accessible for sampling: analysis of EVs isolated from easily accessible biomaterials, such as blood, can give a glimpse into the condition of organs and tissues it is in contact with.

In line with the above, EVs became increasingly popular candidates in biomarker studies, necessitating the development of EV isolation protocols suitable for various types of body fluids. Among isolation techniques are (i) centrifugation-based ones, such as density gradient centrifugation and differential ultracentrifugation, (ii) techniques based on size of the vesicles, such as membrane filtration and size exclusion chromatography (SEC), (iii) techniques based on immunoaffinity capture of the vesicles, and (iv) techniques based on precipitation of EVs with the use of a polymer, most commonly polyethylene glycol (PEG).^4^ While these techniques each can yield EV enrichment, they show great differences in respect of handling time, equipment requirement, and also in obtained EV quality and quantity.^5^ Consequently, the different EV isolation techniques have advantages and downsides depending on the downstream application the EV samples will be used/analyzed in. Simultaneously to the technical developments, guidelines for the evaluation and reporting on the isolated EVs has been established by the International Society of Extracellular Vesicles (ISEV), emphasizing the importance of multimodal EV characterization.^6^

This protocol provides step-by-step descriptions for size-based isolation with the use of in-house made SEC columns, and following characterization of EVs from human plasma samples. We give a detailed description of the preparation and packing of the SEC columns, including materials, column assembly, equilibration, and quality control steps to ensure reproducibility and optimal EV separation. The vesicles are then characterized in accord with the recommendations described in the guideline “Minimal Information for Studies of Extracellular Vesicles” (MISEV).^7^ This includes particle number and size determination via nanoparticle tracking analysis (NTA), EV morphology characterization via transmission electron microscopy (TEM), and assessment of the presence of EV and non-EV associated protein markers via SDS-PAGE and western blot (WB) analysis.

### Innovation

The protocol presented here combines existing methods for the isolation and characterization of human plasma-derived EVs into a concise, updated protocol. An increasing number of studies in the recent years show that EVs acquire a biomolecular corona (BC) via association with, and absorption of biomolecules they become in contact with during their biogenesis, and also after their release in the extracellular space.^8^^,^^9^ The recognition of the importance of the BC in the functionality of EVs,^10^ and its potential as a source of disease markers^11^ brought to the forefront isolation techniques preserving the EV BC. SEC-based EV isolation is a gentle, size-based particle separation method, which involves no denaturing or high-speed centrifugation steps which generate shearing forces, thus SEC isolation of EVs causes less surface disruption and a better maintained native EV structure.^12^^,^^13^ The SEC-based EV isolation approach we provide here requires neither special laboratory equipment nor specialized laboratory personnel training. The EV-analysis methods described in this protocol adhere to the current guidelines and provide instructions for a multimodal EV quality control approach, crucial prior to downstream EV-based assays.

### Institutional permissions

All blood samples processed in this protocol were collected at the University Clinic of Erlangen, Department of Molecular Neurology. Plasma samples were stored at the Biobank of the Department approved by the ethics committee of the Friedrich-Alexander-Universität Erlangen-Nürnberg, Erlangen, Germany under the ethical license #259_17B. Written informed consent was obtained from all participants involved in the study.

***Note:*** Use and handling of human samples requires ethical permissions and should strictly follow the rules of appropriate authorities regarding sample collection, biobanking, and processing.

### Plasma sample collection and processing for storage

**Timing: 1.5–2 h**

1. Collect blood samples from donors following obtaining informed consent.

**Figure 1:**
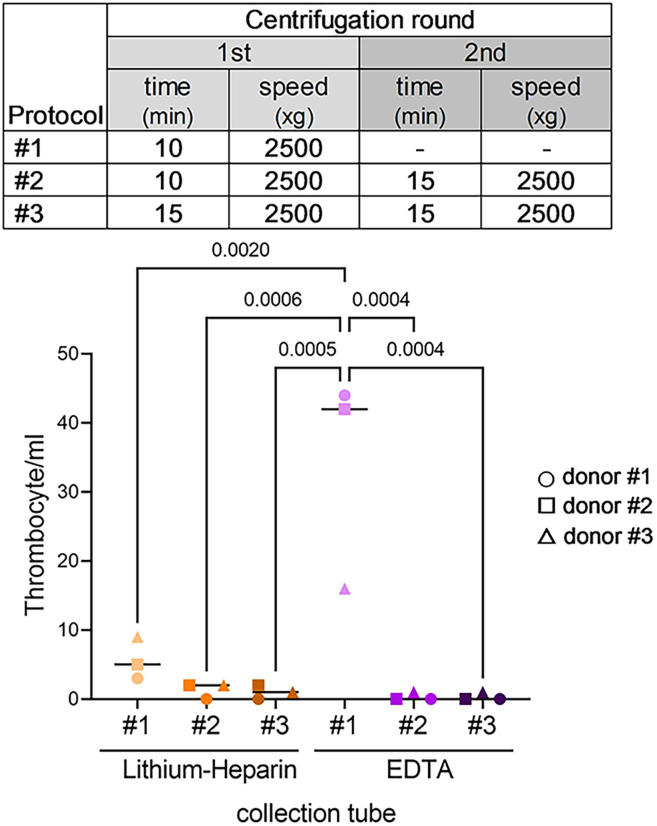
Effect of anticoagulant used and number of centrifugation steps on residual platelet amount in plasma preparations Results of plasma preparation protocols #1, #2, and #3 applied to blood samples collected into Lithium-Heparin and EDTA containing tubes are compared in respect of the number of residual platelets. Thrombocyte concentration was measured on an impedance-based automated hematology analyzer. A one step centrifugation protocol (10 min at 2,500 × *g*) with EDTA-blood samples yields significantly higher residual platelet amount than it is the case with blood samples collected in Lithium-Heparin containing tubes. A second round of centrifugation is beneficial for removing residual platelets from plasma preparations. While this effect results only in a slight trend of platelet number decrease in the case of samples collected in Lithium-Heparin containing tubes, it is more pronounced when using EDTA containing blood collection tubes. (Statistical test: one-way ANOVA followed by Tukey's multiple comparison test. The horizontal lines on the graph indicate the median of the three data points.)

***Note:*** For this study, blood samples were collected by trained study nurses from the cubital vein with the use of a 21G needle in Lithium-Heparin containing blood collection tubes (Sarstedt, #02.1065). Depending on planned downstream analyses however, the use of collection tubes containing other anticoagulants such as EDTA might be necessary. A comparison of plasma purity depending on anticoagulants and number of centrifugations steps used for plasma preparation is given in Figure 1.

In the further steps of this protocol 1.5 ml plasma, equivalent of approximately 3-4 ml blood was used for one EV sample isolation. The input plasma amount however can be decreased if necessary – with the protocol described below we have successfully used 0.5 ml plasma for EV sample preparation as well.

In the experiments described here plasma samples obtained from the institutional biobank were used. The biobank compiles samples of healthy controls, patients with Parkinson's disease, and atypical parkinsonism. As the focus of the experiments presented in this protocol is to demonstrate the technical aspects and reproducibility, independent of disease type, as exclusion and inclusion criteria parameters concerning the collection, handling, and storage of the plasma samples were applied. These parameters are outlined in section “plasma sample collection and processing for storage”.

2. Prepare plasma sample for storage.
  a. Transport tubes at 20°C–24°C to laboratory.
  b. Centrifuge blood samples for 10 min at 2,500 × *g* at 20°C–24°C. A HERAEUS Multifuge 1S-R with swing-out Sorvall 75902000 rotor or equivalent with brake set to max (9) is suitable for this step.
    ***Note:*** For the establishment of this protocol plasma preparation was always started 25–35 min following blood collection.
  c. With the use of P1000 pipette transfer supernatant (the plasma fraction) to a 15 ml conical centrifuge tube (Sarstedt #62.554.502).
    ***Note:*** Repeating the centrifugation step can improve plasma purity by decreasing the number of residual platelets (Figure 1). Plasma samples used in this study were prepared by a single step centrifugation prior to sample freezing.
    If plasma is prepared from multiple blood collection tubes of the same donor, combine all plasma preparations in one 15 ml conical centrifuge tube (Sarstedt #62.554.502) before distributing aliquots.
  d. Invert tube 10-times.
  e. Transfer 1 ml aliquots in 1.5 ml low protein binding microtubes.
3. Store plasma aliquots at −80°C until further use.

***Note:*** Make sure to adhere to institutional policies regarding sample storage. Store the aliquots in an anonymized-manner, with decoding possible only by personnel with granted access.

***Note:*** In order to improve reproducibility of the findings of studies analyzing blood-derived EVs, we call attention to data recording and reporting as suggested in the Minimal Information for Blood EV research (MIBlood-EV).^14^ This tool has been created by the International Society of Extracellular Vesicles (ISEV) Blood EV Task force to facilitate the reproducibility of studies using blood-derived EVs by the recording and reporting of pre-analytical protocol information and assays used to assess the quality of EV preparations.

## Key resources table

**Table:** 

REAGENT or RESOURCE	SOURCE	IDENTIFIER
**Antibodies**		

Rabbit anti-CD9 monoclonal antibody (working dilution: 1:1000)	Cell Signaling Technology	13403S
Rabbit anti-albumin polyclonal antibody (working dilution: 1:2000)	Antibodies-online	ABIN94851
Rabbit anti-Alix monoclonal antibody (working dilution: 1:500)	Cell Signaling Technology	92880S
Rabbit anti-ApoB polyclonal antibody (working dilution: 1:1000)	Proteintech	20578-1-AP
Mouse anti-Flotillin-1 (Flot1) monoclonal antibody (working dilution: 1:250-1:500)	BD Biosciences	610820
Donkey (polyclonal) anti-rabbit IgG (H+L) antibody conjugated with IRDye 800CW (working dilution: 1:10 000)	Li-Cor Biosciences	926-32213
Donkey (polyclonal) anti-mouse IgG (H+L) antibody conjugated with Alexa Fluor™ 680 (working dilution: 1:10 000)	Invitrogen	A10038

**Biological samples**		

Human plasma samples	Biobank of the Department of Molecular Neurology, University Clinic of Erlangen (ethical license #259_17B)	N/A

**Chemicals, peptides, and recombinant proteins**		

Sepharose™ CL-2B	Cytiva	17-0140-01
Dulbecco′s Phosphate Buffered Saline	Sigma-Aldrich	D8537-500ML
Pierce BCA protein assay kit	Thermo Scientific	23225
Pierce Micro BCA protein assay kit	Thermo Scientific	23235
NuPAGE™ LDS sample buffer (4x)	Invitrogen	NP0007
NuPAGE™ sample reducing agent (10x)	Invitrogen	NP0009
PageRuler Plus Prestained Protein Ladder	Thermo Scientific	26619
MES anhydrous BioChemica	PanReac AppliChem ITW Reagents	A0689
Tris base	Sigma	T1503
Sodium dodecyl sulfate (SDS)	Merck	8.17034.1000
EDTA	PanReac AppliChem ITW Reagents	A5097
Glycine	PanReac AppliChem ITW Reagents	A1067
Methanol	Supelco	1060092500
Paraformaldehyde (PFA)	VWR	28794.295
PBS buffer (10x) powder	PanReac AppliChem ITW Reagents	A0965,9010
Ponceau S solution	Sigma-Aldrich	P7170-1L
Intercept blocking buffer	Li-Cor Biosciences	927-60001
Intercept antibody diluent	Li-Cor Biosciences	927-65001
Sodium chloride (NaCl)	Supelco	1.06404.5000
Tween-20	PanReac AppliChem ITW Reagents	A4974
Gelatine from cold water fish	Sigma-Aldrich	G7041-500G
Coomassie brilliant blue (CBB) - G250	PanReac AppliChem ITW Reagents	A3480
Aluminium sulfate hydrate	Carl Roth GmbH	3731.1
Ethanol (96%)	Carl Roth GmbH	T171.4
Ortho-phosphoric acid	Carl Roth GmbH	6366.1
Uranyl Acetate	Electron Microscopy Sciences	22400
2-Propanol (Isopropanol) (70%)	Carl Roth GmbH	CN09.4

**Software and algorithms**		

Excel	Microsoft	https://www.microsoft.com/en-us/microsoft-365/excel
SMART Control reader control software	BMG Labtech	https://www.bmglabtech.com/en/microplate-reader-software/
MARS Microplate Data Analysis Software	BMG Labtech	https://www.bmglabtech.com/en/microplate-reader-software/

**Other**		

Plasma collection tubes	Sarstedt	02.1065
21G blood collection butterfly cannula	Sarstedt	85.1638.235
Heraeus Multifuge 1S-R centrifuge	Thermo Scientific	75004331
Sorvall Heraeus swinging bucket centrifuge rotor	Thermo Scientific	75902000
50 ml transparent conical centrifuge tube	Sarstedt	62.547.254
15 ml transparent conical centrifuge tube	Sarstedt	62.554.502
1.5 ml low-binding microtube	Sarstedt	72.706.600
P1000 pipette tip	Np Green tip	06-379-2018
Seropipette (10, 25, or 50 ml)	Sarstedt	86.(1254/1685/1256).001
Emerald™ Syringe 10 mL	BD	307736
Stericup Quick Release-GP Sterile Vacuum Filtration System	Millipore	S2GPU05RE
20 μm pore size nylon filter	Millipore	2002500
Combi Stopper	Braun	4495152
Parafilm M	Amcor	PM996
Microcentrifuge	Eppendorf	5430R
Microcentrifuge rotor	Eppendorf	FA-45-24-11 HS
1.5 ml microtube	Sarstedt	72.690.009
96-well round bottom microplate	Sarstedt	82.1582
Empore™ sealing film	Supelco	66881-U
Clariostar Plus microplate reader	BMG Labtech	N/A
Thermomixer comfort, 2 ml block thermostat	Eppendorf	5355
MiniStar Galaxy Microcentrifuge	VWR	C1413
Bolt™ 4-12 %, Bis-Tris, 1.0 mm, Mini-Protein-Gel	Invitrogen	NW04125BOX
Mini Gel Tank	Invitrogen	A25977
Mini-Blot-modul	Invitrogen	B1000
Immobilon®-FL PVDF Membrane	Millipore	IPFL00010
Whatman paper	Cytiva	3030-917
pH-indicator strips	Roth	92120
Rotilabo folded cellulose filter, type 600P	Carl Roth	CA25.1
50 ml tube, brown	Sarstedt	62.548.304
Milli-Q water purification system	Millipore	QTUM000EX
Odyssey M Imaging system	Li-Cor Biosciences	3350-00
Amicon ultra centrifugal filter, 10 kDa MWCO	Millipore	UFC5010
Amicon ultra centrifugal filter, 100 kDa MWCO	Millipore	UFC5100
Zetaview device	Particle Metrix	PMX-110
PS beads standard dispersion	Particle Metrix	700074
Injekt-F 1 ml syringe	Braun	9166017V
Carbon film covered copper grids with 300 mesh	Electron Microscopy Sciences	CF300-CU-50
Negative Glow Discharger	Quorum Sputter Coater	SC7620
Inverse forceps; reverse action tweezers (fine tip, curved, stainless steel)	N/A	N/A
Soft tissue paper	Kleenex	N/A
20-24°C Transmission Electron Microscope operating between 80-120 kV acceleration voltage and equipped with digital camera	Jeol	1400Plus

## Materials and equipment

**Table:** MES running buffer (10x):

Reagent	Final concentration	Amount
MES	500 mM	97.6 g
Tris base	500 mM	60.6 g
SDS	1 %	10 g
EDTA	10 mM	3 g
Milli-Q H_2_O	N/A	1000 mL
**Total**	**N/A**	**1000 mL**

Store at 20–24°C up to 6 months.

**CRITICAL:** SDS and EDTA are hazardous to health and environment. Handle and dispose according to the appropriate safety regulations.

***Note:*** Prepare 1x MES running buffer on day of experiment by adding 100 mL 10x MES running buffer to 900 mL Milli-Q H_2_O.

**Table:** Transfer buffer (10x):

Reagent	Final concentration	Amount
Glycine	769 mM	57.7 g
Tris base	95 mM	11.6 g
Milli-Q H_2_O	N/A	1000 mL
**Total**	**N/A**	**1000 mL**

Store at 20–24°C up to 6 months.

**Table:** Transfer buffer (1x):

Reagent	Final concentration	Amount
10x transfer buffer	1x	100 ml
Milli-Q H_2_O	N/A	700 ml
Methanol	20%	200 mL
**Total**	**N/A**	**1000 mL**

***Note:*** Prepare on the day of experiment. When preparing, keep order of components as listed to avoid precipitate formation.

**CRITICAL:** Methanol is a flammable, toxic substance. When handling and disposing follow appropriate safety regulations.

4% paraformaldehyde (PFA) solution:

• Heat 900 ml of distilled water in a beaker in the microwave (do not boil).
• Add a magnetic stirring bar, and place the beaker on a magnetic stirrer under a fume hood.
• Add 40 g PFA while stirring.
• Let stir for 15 to 30 min with the heating block set to 70°C.
• Add a few drops (approx. 100 μl) of 10M NaOH until the solution becomes clear.
• Add 100 ml 10x phosphate buffered saline (PBS).
• Test the pH with indicator paper, if necessary adjust so that it is pH 7.4.
• Let the solution cool to 20–24°C, filter (Rotilabo folded cellulose filter, type 600P, Carl Roth #CA25.1), and aliquot in 10 or 50 ml conical centrifuge tubes.

***Note:*** Store aliquots at −20°C up to 6 months. Once thawed, store at 4°C up to 2 weeks.

***Note:*** On the day of experiment prepare 0.4% PFA solution by adding 1 mL 4% PFA to 9 ml Milli-Q H_2_O in 50 ml brown conical centrifuge tube.

**CRITICAL:** PFA is an irritant, sensitizer, flammable, toxic, classified as probable carcinogenic substance. PFA and its container must be disposed of as hazardous waste. When handling and disposing follow appropriate safety regulations.

**Table:** Tris-buffered saline (TBS; 10x):

Reagent	Final concentration	Amount
Tris base	1M	121.1 g
NaCl	1.5M	87.6 g
Milli-Q H_2_O	N/A	1000 mL
**Total**	**N/A**	**1000 mL**

Adjust pH to 7.4. Store at 20–24°C up to 6 months.

**Table:** Tris-buffered saline containing 0.1% Tween-20 (TBS-T):

Reagent	Final concentration	Amount
10x TBS	1x	100 mL
Tween-20	0.1%	1 mL
Milli-Q H_2_O	N/A	1000 mL
**Total**	**N/A**	**1000 mL**

Store at 20–24°C up to 6 months.

### 2% fish gelatine in TBS-T

In a glass bottle place a magnetic stirring rod, add 500 mL TBS-T and 10 g fish gelatine powder. Stir on magnetic stirrer while heating at 20–24°C. When the fish gelatine is completely dissolved, aliquot in 50 ml conical centrifuge tubes. Store at −20°C up to 6 months.

Prior to use, thaw overnight at 4°C. Once thawed, store at 4°C up to 2 weeks.

**Table:** Coomassie brilliant blue (CBB) colloidal stain:

Reagent	Final concentration	Amount
CBB-G250	0.02%	0.2 g
Aluminium sulfate hydrate	5%	50 g
96% Ethanol	10%	100 mL
ortho-phosphoric acid (100 %)	2%	23.5 mL
Milli-Q H_2_O	N/A	876.5 mL
**Total**	**N/A**	**1000 mL**

To prepare the solution, place a glass bottle containing a magnetic stirring bar on a magnetic stirrer and first dissolve the aluminium sulfate in Milli-Q H_2_O. Add 100 ml ethanol, homogenize, and mix in the CBB G-250. Once the aluminium sulfate dissolved, add the ortho-phosphoric acid. Fill up with Milli-Q H_2_O to a final volume of 1000 mL. In the resulting dark greenish-blue staining solution you will notice floating color particles, which are the colloidal form of Coomassie, and should not be filtered out. Seal the bottle tightly and store at 20–24°C up to 6 months.

***Note:*** An aliquot of the staining solution can be re-used for multiple gels. If the staining potential is lost, dispose in designated waste collector.

**CRITICAL:** Note that handling and disposing CBB-G250, ethanol, aluminium sulfate-hydrate, and phosphoric acid must be done in accord with the appropriate safety regulations concerning these reagents.

### 2% aqueous uranyl acetate solution

• Weigh 0.1 g uranyl acetate into a 15 ml conical centrifuge tube.
• Add 5 ml of Milli-Q H_2_O.
• Cover tube with aluminum foil.

***Note:*** One layer of aluminum foil is sufficient and allows ultrasound waves to penetrate.

• Place in ultrasound bath for >10 min. If uranyl acetate is not fully dissolved place in ultrasound bath again for 10 min.
• Centrifuge at 1,000 × *g* for 5 min.
• Save supernatant (should be yellow with a touch of green) for further use and discard the pellet (if there is any).
• Aliquot and store in the refrigerator at 4°C and shield from light (use e.g., dark microtubes or tubes covered with aluminum foil)

**CRITICAL:** Uranyl acetate is a α-radiator and poisonous if ingested, inhaled, or in contact with mucous surfaces. For safe handling refer to local regulations and purchase restrictions.

## Step-by-step method details

### Preparation of size-exclusion chromatography columns

**Timing: 4.5**–**5 h**

Protocols for EV-SEC were adapted from.^15^

**Figure 2:**
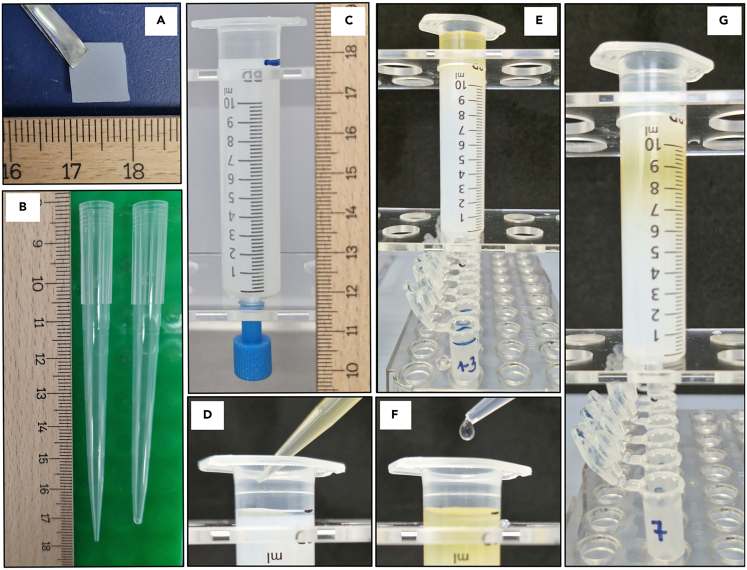
SEC column preparation, sample loading, and fraction collection (A) An approximately 1x1 cm piece nylon filter is used to cover the syringe exit to prevent Sepharose from exiting. (B) A cut P1000 pipette tip is used when pipetting Sepharose when preparing the columns. (C) SEC column on column holder, with closed syringe exit. (D) Plasma sample is added when only a 1-2 mm DPBS layer is present on top of the column matrix to minimize sample dilution. The sample is added carefully, leaving the top layer of the column matrix undisturbed. (E) Immediately start collecting fractions of 500 μl volume each into 1.5 ml microtubes. Collect the first 3 ml of the sample by filling two 1.5 ml tubes (this is the void volume corresponding to the fist 6 fractions. This can be discarded.). (F) Add the first 500 μl aliquot of DPBS only when the sample nearly completely entered the column matrix in order to avoid sample dilution. Never let the column run dry, make sure to always have a layer of liquid covering the surface of the column matrix. When adding DPBS, be very careful not to disturb the top layer of the column matrix. Using a cut P1000 pipette tip may help more gentle, drop-wise buffer loading. (G) From fraction 7 onwards each 500 μl fraction is collected in a separate microtube.

1. Equilibrate Sepharose CL-2B (Cytiva, #17-0140-01) with DPBS
  **CRITICAL:** Use 0.2 μm filtered DPBS in every step where it will be in contact with the sample to avoid sample contamination with nanoparticles.
  a. Transfer up to 30 ml Sepharose CL-2B into sterile 50 ml conical centrifugal tube.
    ***Note:*** In the current protocol one column contains 12 ml set Sepharose CL-2B.
  b. Let Sepharose CL-2B set at 20–24°C.
  c. Once the beads settled, carefully remove and discard supernatant with the use of a seropipette.
  d. Add filtered DPBS equal volume to the settled Sepharose CL-2B and invert in hand 10-times/until there is no Sepharose left set in the bottom of the tube.
  e. Let Sepharose CL-2B settle at 20–24°C.
  f. Once the beads settled, carefully remove and discard DPBS with the use of a seropipette.
  g. Repeat steps (d) to (f) two more times
  h. Add equal volume of filtered DPBS to the set Sepharose and invert in hand 10-times/until there is no Sepharose left set in the bottom of the tube.
2. Assemble SEC columns
  a. Remove plunger of 10 mL syringe (BD 10 ml Emerald, #307736).
  b. Mark 6.5 cm from the bottom of the syringe.
  c. Cut an approximately 1x1 cm piece of a nylon filter (Millipore, cat.no.: 2002500; 20 μm pore size) (Figure 2A) and place in the syringe to cover the syringe exit.
    ***Note:*** To minimize potential sample contamination, clean scissors/blade and forceps used for cutting and handling the filter with 70% isopropanol prior to use.
  d. Place the syringe on a holder with a cannister under to collect the flow-through.
  e. Carefully pipette approximately 2 ml filtered DPBS on top of the nylon filter.
    ***Note:*** Ensure that the filter lays smoothly on the syringe bottom covering the syringe exit. See troubleshooting problem 1.
  f. Prepare the Sepharose CL-2B column by pipetting 1 ml aliquots of the matrix into the column until the desired settled volume (6.5 cm height) is reached.
    ***Note:*** In order to prevent crushing Sepharose CL-2B beads while pipetting them, use cut end pipette tips for preparing the column. For this cut off 5mm from the tip of a P1000 pipette tip before use (Figure 2B).
    **CRITICAL:** Prior to starting, make sure to let Sepharose CL-2B and DPBS to equilibrate to ambient temperature to avoid bubble formation. See troubleshooting problem 2.
    **CRITICAL:** Do not let the column run dry, always ensure that the matrix surface is covered with filtered DPBS.
  g. Once desired column height is reached, close the syringe exit (Braun Combi Stopper, #4495152) (Figure 2C), cover top with parafilm, and store at 4°C for 16h.
    **CRITICAL:** An even and perfectly horizontal column surface is necessary to ensure proper sample separation.
    **CRITICAL:** Make sure to always have a layer of approximately 5 mm DPBS on top of set Sepharose CL-2B, and keep the columns in an upright position during storage.

### SEC of plasma EVs

**Timing: 1 h per sample**

3. Thaw two 1 ml plasma aliquots on ice.

***Note:*** All samples used in this protocol were thawed once, just before further processing.

4. Remove aggregates formed during freezing and thawing. See troubleshooting problem 3.
  a. Centrifuge at 2,500 × *g* for 15min at 4°C.
  b. Without disturbing the pellet, transfer supernatant to new low protein binding 1.5 ml microtube.
  c. Repeat centrifugation at 2,500 × *g* for 15 min at 4°C.
  d. Transfer supernatant to new low protein binding 1.5 ml microtube.
5. Transfer 1.5 ml plasma sample on top of SEC column (Figure 2D).

**CRITICAL:** Prior to use, let SEC column and DPBS equilibrate to ambient temperature in order to avoid bubble formation. See troubleshooting problem 2.

***Note:*** To minimize sample dilution, load plasma sample when only a 1-2 mm DPBS layer is present on top of the column matrix. When adding plasma sample and later DPBS, be very careful not to disturb the top layer of the column matrix. Always aim to load sample and DPBS on top of column by creating an even liquid layer.

6. Immediately start collecting fractions of 500 μl volume each into 1.5 ml microtubes (Figure 2E). Place the fraction collected immediately on ice.

**CRITICAL:** Never let the column run dry, make sure to always have a layer of liquid covering the surface of the column matrix. Add the first 500 μl aliquot of DPBS only when the sample nearly completely entered the column matrix in order to avoid sample dilution (Figure 2F). Add this smaller volume (500 μl) DPBS two more times, then the volume of following steps can be increased to 1 ml.

***Note:*** Collect 3 ml of the sample by filling two 1.5 ml tubes (this is the void volume corresponding to the fist 6 fractions. This can be discarded.). Following this, collect 500 μl of each fraction separately (Figure 2G). EVs elute typically in fractions 8 to 12.

**Pause point:** Fractions can be stored at −20°C for up to 7 days prior to further processing.

***Note:*** To avoid repeated freeze-thaw cycles, we recommend taking 35 μl aliquots of each fraction for protein concentration determination and immunoblotting.

### Determining protein concentration of SEC fractions with BCA assay

**Timing: 1 h**

**Figure 3:**
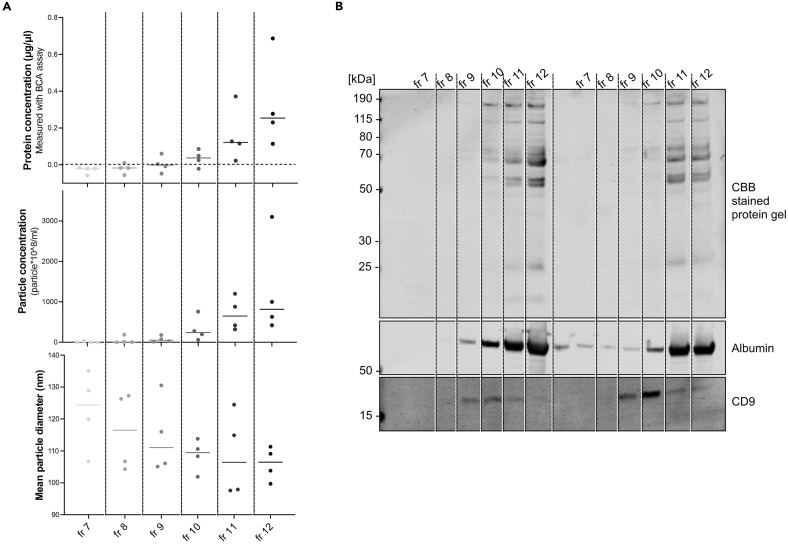
Protein concentration, SDS-PAGE and Western blot, and nanoparticle tracking analysis of plasma SEC fractions (A) Protein concentration is increasing in later fractions of SEC separated plasma samples (top graph). In parallel with this, the particle concentration measured with NTA also shows an increase with increasing fraction number (middle graph), while particle size decreases (bottom graph). Each data point on the graph represents an individual sample, the horizontal lines indicate the median of the four data points. Protein concentration, particle concentration and diameter values in fractions collected from four SEC separations are shown. (B) SDS-PAGE and WB analysis of fractions of two representative EV-SEC separation. Though CD9 signal is visible from fraction 8 to fraction 12, it is the strongest in fractions 9 and 10, indicating these to be the richest in EVs. On the other hand, the amount of both total proteins (visible on Coomassie brilliant blue stained protein gel) and albumin are the highest in later fractions, indicating that the high protein concentration of fractions 10-11-12 is mainly due to the higher amount of non-EV related proteins. Vertical guide lines were overlaid on the image to help orientation between lanes.

7. Prepare bovine serum albumin (BSA) standards ranging from 2 mg/ml to 0.03125 mg/ml by diluting BSA stock (provided with Pierce BCA protein assay kit, Thermo Scientific, #23225) in DPBS. A convenient way of preparing the standards is making a 6-step 2-fold dilution series. For this
  a. Pipette 60 μl from the 2 mg/ml BSA to a 1.5 ml microtube and mark as tube 1.
  b. Add 30 μl DPBS into six 1.5 ml microtubes each, and mark these as tube 2–7.
  c. Transfer 30 μl from tube 1 to tube 2 and vortex.
  d. Transfer 30 μl from tube 2 to tube 3 and vortex.
  e. Continue until tube 7 is prepared.
8. In a 96-well plate (Starstedt, #82.1582) aliquot 10 μl from each standard dilution in technical replicates, and 10 μl from each collected SEC fractions.
9. Prepare reaction mastermix by mixing 100 μl Reagent A with 2 μl Reagent B (Pierce BCA protein assay kit, Thermo Scientific, #23225) per reaction.
10. With a multipipette, add 100 μl reaction mastermix to each well.
11. Cover plate with sealing film (Empore sealing film, Supelco, #66881-U) and incubate at 37°C for 30 min.
12. Remove plate from incubator and let it cool to 20–24°C (approximately 3–5 min).
13. Remove sealing film and measure absorbance at 562 nm in a microplate reader (Clariostar Plus, BMG Labtech).
14. With the absorbance values of the BSA standards generate standard curve, and determine protein concentration of the fractions (Figure 3A).

**Figure 4:**
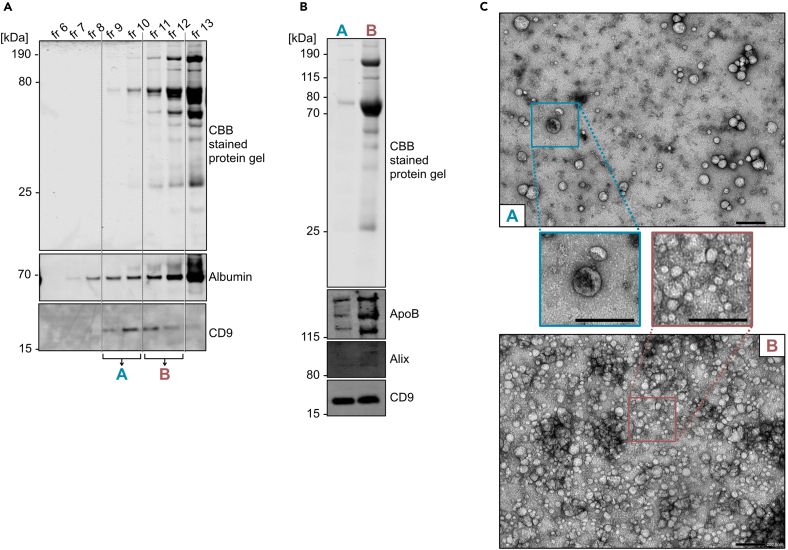
Immunoblot- and transmission electron microscopy analysis of free-protein poor and rich EV samples (A) SDS-PAGE and WB analysis of a representative EV-SEC sample. Signal intensities of the positive EV marker CD9 and the presence of free, non-EV related proteins show an inverse relation. While CD9 signal peaks in fractions 10 and 11, total protein amount (visible on Coomassie brilliant blue stained protein gel) and albumin signal intensity show a gradual increase, becoming more pronounced starting from fraction 11. Vertical guide lines were overlaid on the image to help orientation between lanes. (B) SDS-PAGE and WB analysis of samples prepared via combining and concentrating fractions 9-10, and 11-12 (resulting in samples A and B, respectively. In each case 440 μl of fractions were used and concentrated 11-fold with the use of a 100kDa cutoff Amicon centrifugal filter, to a final sample volume of 80 μl. 20 μl sample A and B was loaded on SDS-PAGE.) demonstrates that while signal intensities of the positive EV markers CD9 and Alix are comparable between the two samples, the total protein content (visible on Coomassie brilliant blue stained protein gel), and the lipoprotein associated ApoB signal show prominent increase in sample B as compared to sample A. The high number of vesicles visible when analyzing sample B via TEM (C) can be misleading, since considering the strong ApoB signal detected with immunoblotting, a large number of the particles are likely lipoproteins and other aggregates. Scale bar: 200 nm.

### SDS-PAGE and WB analysis of SEC fractions

**Timing: 2 days**

The following steps describe a protocol for immunoblotting for EV-markers. This technique is used to assess the EV and free protein content of the fractions.

***Note:*** The SDS-PAGE and WB protocol described here specifies laboratory equipment, reagents, and experimental conditions as an example of a validated setup yielding reproducible readouts. However, these parameters are not exclusive or universally required for successful results. Alternative types of gels, buffers, blotting conditions, and antibodies may also be suitable. The use of these requires appropriate optimization and validation under the specific experimental conditions of the user.

15. Prepare samples for loading on SDS-PAGE.
  a. Mix 20 μl from each selected fraction with 7 μl 4x sample loading buffer (NuPAGE LDS sample buffer, Invitrogen, #NP0007) and 3 μl 10x reducing agent (Invitrogen, #NP0009).
    ***Note:*** Depending on the antibody of choice, omitting the reducing agent may be required.
  b. Heat samples in block thermostat (Eppendorf Thermomixer Comfort, #5355) for 10 min at 70°C with 300 rpm shaking.
  c. Spin down samples with benchtop microcentrifuge for 1 min (MiniStar Galaxy Microcentrifuge, VWR, #C1413).
16. Separate proteins with SDS-PAGE.
  a. Load samples and marker (Thermo Scientific, PageRuler Plus Prestained Protein Ladder, cat.no.: 26619; 2.5 μl) on Bis-Tris gel (for establishing this protocol NuPAGE pre-cast, 1mm, 4–12% Bolt Bis-Tris Plus gels were used (Invitrogen, #NW04125BOX)).
  b. Run in 1x MES running buffer in mini-gel tank (Invitrogen, A25977) at 80V for 10 min, and continue at 120V, for a total of approximately 1.5 h.

**Figure 5:**
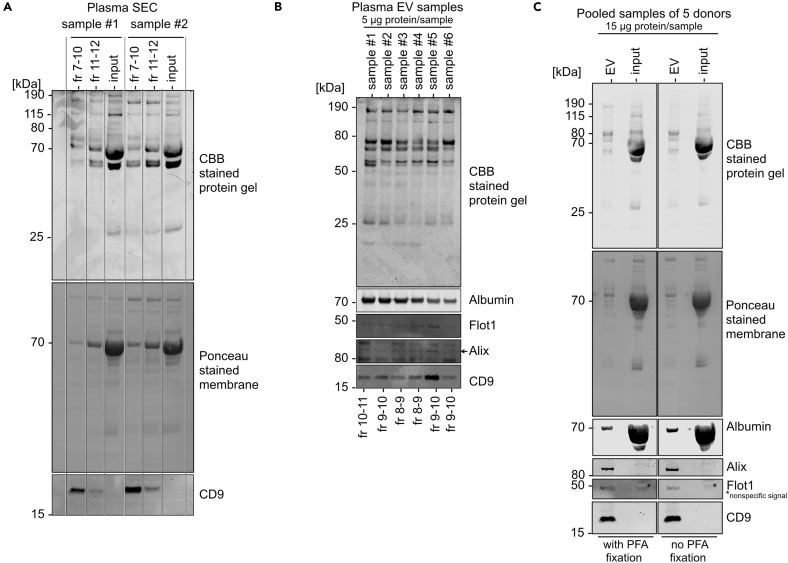
Immunoblot analysis of concentrated EV samples (A) SDS-PAGE and WB analysis of concentrated fractions (fraction concentration was performed using 10 kDa cutoff centrifugal filters) from two representative EV-SEC. In case of each sample 5 μg protein was loaded (protein concentration was determined at A280). For both samples, concentrates of early SEC fractions (fr 7-10) yielded stronger CD9 signal as compared to later fractions (fr 11-12) and plasma input samples. In contrast with that, higher molecular weight proteins show enrichment in the latter two sample types as compared to fr 7-10 EV samples (total protein staining with Coomassie brilliant blue (CBB) and Ponceau S stain of SDS-PAGE and membrane, respectively). Vertical guide lines were overlaid on the image to help orientation between lanes. (B) SEC was performed on plasma samples of six donors separately. Fractions of interests (indicated in the bottom of the blot) were selected based on the results of the WB analysis of the individual fractions. These fractions were then combined and concentrated (using 100 kDa cutoff centrifugal filters), yielding an average 1.03∗10^10^ particle/1.5 ml plasma. (C) Comparison of total protein pattern, presence of intravesicular, and membrane-associated EV markers, and albumin in EV-enriched and input plasma samples. EV samples were prepared following the SEC protocol described here. Fractions of interest were selected based on the protein concentration of the individual fractions determined via BCA. For the concentration of SEC fractions 100 kDa cutoff Amicon filters were used. Samples of equal protein content of five donors were pooled, and 15 μg protein of EV and input samples were loaded in duplicates onto the gradient gel. Following transfer the membrane was cut in two, and one half of it was fixed in 0.4% PFA (left panel), while for the other no fixation was performed (right panel), in order to assess the effect of PFA fixation on the detection of proteins of interest. The strong intravesicular- and membrane-marker signals (Alix, CD9, Flot1) in the EV samples and lack of these in the input samples demonstrate successful enrichment. The prominent decrease in albumin signal and in the amount of other high molecular weight proteins (as seen on total protein stained protein gel and membrane) demonstrate successful separation of EV-rich and protein-rich samples. PFA fixation of the membrane did not affect the detection of neither of the proteins investigated here.
17. Transfer proteins to membrane and probe for negative and positive EV markers.
  a. Transfer proteins onto PVDF membrane (0.45 μm pore size, Immobilion-FL, #IPFL00010) using mini-blot-module (Invitrogen, #B1000) placed in mini-gel tank filled with 1x Transfer buffer containing 20% Methanol. Transfer for 1 hour 10 min, at 20–24°C, at constant 30 V (will be at approx. 230 mA).
  b. Remove membrane from the blotting chamber and place in a 50 ml brown conical centrifuge tube (Sarstedt, #62.548.304).
    ***Optional:*** Perform Coomassie Brilliant Blue (CBB) staining of protein gel to visualize residual protein following blotting.
    i. Transfer protein gel to an approximately 11 x 8 cm incubation box and add Milli-Q H_2_O so that the gel is completely covered.
    ii. Incubate at 20–24°C for 1 hour on platform shaker (Lauda).
    iii. Discard water and add CBB solution to cover the gel.
    iv. Incubate for 16 h at 20–24°C with constant shaking.
    v. Remove CBB stain add Milli-Q H_2_O.
    vi. For destaining, change water 3 times, in 2-3-h intervals.
    vii. The following day scan with Odyssey M imaging system (Li-Cor Biosciences).
    ***Optional:*** Fixation of WB membranes using PFA has been shown to increase signal intensity and thus detectability of certain small sized, highly soluble proteins, such as alpha-synuclein.^16^^,^^17^ With the antibodies used in this protocol we did not observe any difference in detectability depending on the presence or lack of PFA fixation (Figure 5C). Nonetheless, the use of this step with other antibodies need validation to exclude potential epitope masking.
    i. Fix membrane with 0.4% PFA (10 ml/membrane) by rotating for 20 min at 20–24°C.
    ii. Wash membrane 3 times with Milli-Q H_2_O, each time incubating for 5 min at 20–24°C under constant rotation.
      ***Note:*** Discard PFA and contaminated washes in designated PFA waste collector.
  c. Perform total protein stain on membrane with Ponceau S solution.
    ***Note:*** Other removeable total protein stains (e.g. Revert™ 520 Total Protein Stain, Li-Cor BioSciences, #926-10010) are possible alternatives for this step.
    i. In a clean, approximately 11 x 8 cm incubation box add 7 ml Milli-Q H_2_O and 3 ml Ponceau S solution (Sigma-Aldrich, #SLCQ5486).
    ii. Place membrane in solution, and with continuous gentle rocking in hand, incubate until protein bands appear.
    iii. Discard stain, add Milli-Q H_2_O, and let incubate for 1-2 min with continuous gentle rocking in hand.
    iv. Discard the used and add fresh Milli-Q H_2_O.
    v. Scan with Odyssey M imaging system (Li-Cor Biosciences).
    vi. De-stain membrane with Milli-Q H_2_O, and place back in 50 ml brown conical centrifuge tube (Sarstedt, #62.548.304).
      ***Note:*** Alternatively transfer buffer can be used to reduce de-staining time.
  d. Block membrane with Intercept blocking buffer (Li-Cor Biosciences, #927-60001, 10 ml/membrane) for 1h at 20–24°C under constant rotation.
  e. Incubate with primary antibody diluted in Intercept antibody diluent (Li-Cor Biosciences, #927-65001) (anti-CD9: Cell Signaling Technology, #13403S; 1:1000 dilution, 16h incubation at 4°C; and anti-albumin: Antibodies-online, ABIN94851, 1:2000 dilution, 1.5h incubation at 20–24°C).
  f. Remove primary antibody and wash membrane with 1xTBS containing 0.1% Tween-20. Wash three times, 10 minutes each, under constant rotation.
  g. Add secondary antibody (IRDye 800CW donkey anti-rabbit IgG, Li-Cor Biosciences, #926-32213; 1:10 000 dilution) diluted in 2% fish gelatin in 1xTBS containing 0.1% Tween-20, and incubate at 20.
    ***Note:*** Instead of the 2% fish gelatin in 1xTBS with 0.1% Tween-20 buffer, Intercept antibody diluent (Li-Cor Biosciences, #927-65001) can also be used as a diluent.
  h. Wash membrane three times with 1xTBS containing 0.1% Tween-20, each time for 10 min under constant rotation.
  i. Scan membrane with Odyssey M imaging system (Li-Cor Biosciences) (Figure 3B). See troubleshooting problem 4.
    ***Note:*** Results of the immunoblotting permit combining fractions in accord with requirements of downstream steps: i.e. combining only those SEC fractions which are positive for EV markers and are low in free proteins (earlier EV marker positive SEC fractions) might result in sample of lower EV yield, but higher purity. Compared to that, EV samples obtained by combining earlier and later EV marker positive SEC fractions irrespective that the latter ones show increased free protein content, might lead to EV samples higher in particle numbers together with possible contaminating proteins. With the SEC-based separation described in this protocol, EV-enriched fractions are typically fractions 8–12. If the presence of non-EV proteins in the sample is not interfering with downstream experiments, SDS-PAGE and WB analysis of the fractions can be omitted.

### Sample concentration

**Timing: 2**–**4 h**

This step describes a protocol for concentrating the EV fraction(s) with Amicon ultra centrifugal filters (Millipore; 10 kDa MWCO #UFC5010; 100 kDa MWCO #UFC5100).

***Note:*** Data indicate that the use of filters with 10 kDa cutoff is favorable in order to reduce EV-loss.^18^ However, the use of 100 kDa MWCO filter units can be beneficial for the removal of excess free proteins in the sample. The protocol described below is compatible for using either of the filters mentioned above. By preparing samples consisting of early (fractions 8–10) and later (fractions 11–12) SEC fractions separately, EV samples with different free protein content can be achieved (Figure 4).

18. Thaw fractions of interest on ice.
19. Pre-rinse membrane.
  a. Add 500 μl DPBS onto filter unit.
  b. Centrifuge at 14,000 × *g* for 10 min at 4°C.
  c. Discard flow-through.

**CRITICAL:** Proceed within 20 min to the next step to prevent the membrane from drying out.

20. Combine and concentrate SEC-fractions.
  a. Add approx. 450 μl fraction onto filter unit.
  b. Centrifuge at 5,000 × *g* for 15–20 min at 4°C.
  c. Discard flowthrough.
  d. Add consequent fraction so that the final volume in the filter unit is 450 μl.
  e. Centrifuge at 5,000 × *g* for 15–20 min at 4°C.
  f. Repeat steps (a) to (e) until desired concentrate volume is achieved.
21. Recover sample.
  a. Place the inverted filter in a new collection tube.
  b. Centrifuge at 1,000 × *g* for 2 min at 4°C.
  c. Transfer concentrate to a labelled low protein binding 1.5 ml microtube.
22. Aliquot samples and store at −80°C.

***Note:*** Define aliquot size based on planned downstream analysis in order to avoid multiple freeze-thaw cycles.

### Nanoparticle tracking analysis

**Timing: 15 min per sample**

The following protocol describes steps for nanoparticle tracking analysis (NTA) with ZetaView NTA device.

***Note:*** Besides ZetaView, other devices are also available for determining EV number and size. For the use of these we refer to manufacturer recommendations (e.g. NanoSight Pro instrument by Malvern Panalytical) together with further optimization and validation steps under the specific experimental conditions of the experimenter.

23. Prepare ZetaView device for measurement.
  a. Turn on ZetaView device.
  b. Turn on connected PC and start ZetaView software.
  c. Prepare standard.
    i. Prepare 1000-fold dilution (“standard 1”) of 100 nm PS beads standard dispersion (Particle Metrix #700074; lot 240820) in autoclaved 0.2 μm filtered Milli-Q H_2_O.
    ii. Dilute “standard 1” further 250-fold in autoclaved 0.2 μm filtered Milli-Q H_2_O to prepare “standard 2”.
      **CRITICAL:** Make sure to mix dilutions well prior to use.
      ***Note:*** Do not use dilutions older than two weeks.
  d. Fill the chamber of the NTA device with 10 ml autoclaved and 0.2 μm filtered Milli-Q H_2_O.
  e. Calibrate instrument and set focus.
    i. With the use of 1 ml syringe (Braun; Injekt-F; cat.no.: 9166017V) inject 1 ml standard 2 solution in the chamber.
    ii. Perform focus calibration.
  f. Rinse instrument with 40 ml 0.2 μm filtered DPBS.
  g. Perform cell quality check, continue only if result is “very good”. If not, rinse with further 20 ml DPBS, and repeat cell quality check.
24. Apply instrument settings.

***Note:*** Laser: 405; Sensitivity: 82; Frame rate: 30; Shutter: 50.

25. Measure EV samples.
  a. Dilute EV samples with 0.2 μm filtered DPBS in 1.5 ml low protein binding microtubes.
    ***Note:*** In our experience for EV concentrates prepared as described in this protocol typically 1:1000-1:5000-fold dilution is required of concentrated EV samples of 30–60 μl final volume prepared from 1.5 ml starting plasma volume.
  b. With the use of a 1 ml syringe, inject 1 ml diluted sample into chamber.
    ***Note:*** Do not forget to add dilution factor in the ZetaView software.
  c. Inspect live-video and detected particle number.
    **CRITICAL:** Ensure that no signs of air bubbles can be observed and the particle number is within the accepted range (50 to 350). See troubleshooting problem 5.
  d. In pop-up window add file name and choose folder where measurement files will be saved.
  e. Start measurement.
  f. Export and save measurement results in designated data folder.

### SDS-PAGE and WB analysis of concentrated EV samples

**Timing: Variable**

In order to demonstrate successful EV enrichment with maintained vesicle integrity throughout the isolation process demonstration of the presence of at least one intravesicular-, and one membrane-associated EV marker is necessary. In this protocol we use SDS-PAGE and WB for the assessment of the presence of Alix and CD9 as an intravesicular and membrane-associated positive EV marker, respectively. For the detection of positive and negative EV markers in the concentrated EV samples (Figure 5) follow steps described in section ‘SDS-PAGE and WB analysis of SEC fractions’, with minor modifications.

***Note:*** Proteomics analysis of EV samples can serve as an alternative approach for EV-marker detection. While this method offers higher sensitivity, it presents with higher costs, and requirements of special equipment, and trained personnel.

26. Load 5-15 μg protein/EV sample (concentration determined e.g., at A280nm or with Pierce Micro BCA assay (Thermo Scientific, #23235)) on SDS-PAGE.
27. Selection of antibodies:
  a. Positive EV markers: Rabbit anti-CD9 monoclonal antibody, Cell Signaling Technology, #13403S, 1:1000, 16h incubation at 4°C; Rabbit anti-Alix monoclonal antibody, Cell Signaling Technology, #92880S, 1:500, 16h incubation at 4°C; Mouse anti-Flotillin-1 (Flot1) monoclonal antibody, BD Biosciences, #610820, 1:250-1:500, 16h incubation at 4°C.
  b. Negative EV-markers: Rabbit anti-albumin polyclonal antibody, Antibodies-online, #ABIN94851, 1:2000, 90 min incubation at 20-24°C; Rabbit anti-ApoB polyclonal antibody, Proteintech, #20578-1-AP, 1:1000, 90 min incubation at 20-24°C.
  c. Fluorescently labelled secondary antibodies: Donkey (polyclonal) anti-rabbit IgG (H+L) antibody conjugated with IRDye 800CW, Li-Cor Biosciences, #926-32213, 1:10 000, 1 h incubation at 20-24°C; Donkey (polyclonal) anti-mouse IgG (H+L) antibody conjugated with Alexa Fluor™ 680, Invitrogen, #A10038, 1:10 000, 1h incubation at 20-24°C.

**Figure 6:**
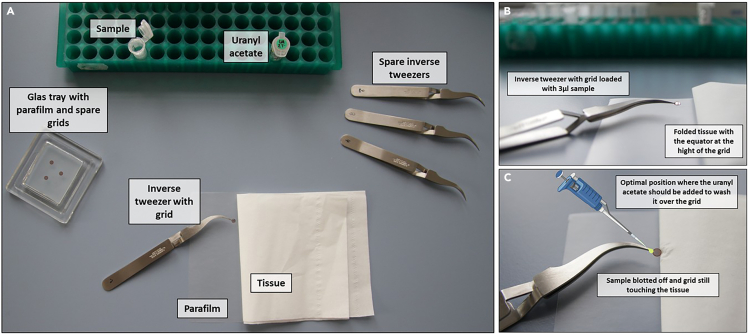
Negative stain procedure (A) Exemplary working space organization (user is left-handed). (B) View from the front to show that the grid and the equator of the tissue fold are at the same height. This ensures optimal blotting results. (C) After blotting excess sample off, 5 μL of 2% uranyl acetate is washed over the grid from the side of the tweezer. Ensure that the grid touches the tissue at all times.

### Transmission electron microscopy sample preparation and imaging

**Timing: Variable**

**Figure 7:**
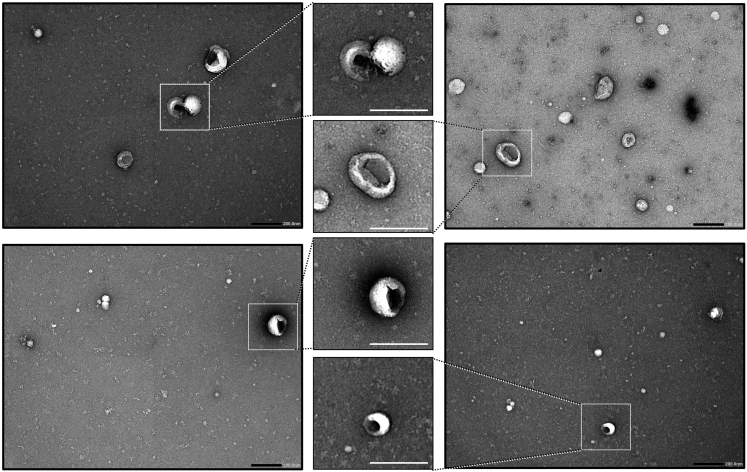
Representative TEM images of plasma-derived EVs SEC was performed with 1.5 ml input plasma, and fractions 7 to 11 were combined and concentrated with 10 kDa cutoff Amicon centrifugal filters. The average concentrate volume of the above samples was 66 μl with a mean particle concentration of 8∗10^10^ particle/ml. For TEM imaging samples were diluted 2-fold. Scale bar: 200 nm.

***Note:*** We call attention to *EV finder*, a recently developed workflow implemented into an ImageJ plugin which enables automated EV size measurement by the analysis of TEM images.^19^

28. Perform negative stain procedure (Figure 6).
  ***Note:*** Negative staining is a rather established method in the field of TEM. There are many different protocols in literature and every researcher has to find their own way. The protocol described below has worked for our purposes and delivers reproducible and consistent results.
  a. Negative glow discharge the TEM grids at 25 mA for 15 sec.
    i. Place grids on a glass tray with parafilm on top.
    ii. Make sure the carbon side is up.
  b. Centrifuge an aliquot of 2% aqueous uranyl acetate solution for 60 sec at 1,000 × *g* to pellet crystals that might have formed.
  c. Take one grid into the inverse forceps while making sure not to exceed the cupper rim of the grid with the tip of the forceps.
  d. Add 3 μl of EV sample (∼1x10ˆ9 EV/ml) to the freshly glow discharged grid.
  e. Incubate for 5 sec.
  f. Touch the side of the grid opposite to the forceps to a fine paper tissue in order to remove excess sample (upon touching, the excess sample should be soaked into the tissue paper).
    ***Note:*** The paper tissue should be folded so that the folded edge/the round side faces the grid. Optimally, the equator of the folded tissue should be at the height of the grid.
  g. Apply 5 μl uranyl acetate solution onto the sample by pipetting it onto the grid at the position where it is held by the forceps.
    **CRITICAL:** Take 5 μl of the 2% uranyl acetate solution from the top of the aliquot and make sure not to resuspend the pellet at the bottom. This will ensure a more evenly distributed staining result without crystal contaminations on the grid. The grid must be touching the tissue and thus the uranyl acetate solution should be soaked into the tissue directly.
  h. Repeat step ‘g'.
  i. Take the forceps with the grid and carefully plot off any uranyl acetate that might have been soaked into the forceps tip.
    ***Note:*** For this, gently touch the edge of a tissue with the position of the forceps where the grid and the tip form an angle.
  j. Air dry the grid for several minutes at 20-24°C.
    ***Note:*** Avoid direct exposure to the sun while drying.
  k. Transfer to TEM.
29. Image EV samples in TEM (Figure 7).
  a. After insertion of grid, a staining gradient should be visible with differently thick staining results.
  b. Start with a medium thick position and image at a magnification where EVs become visible (∼10-15Å pixel length).
  c. EVs appearing with different contrast indicate differences in the thickness of the staining. EVs with a dark rim are characteristic of a thin staining, whereas vesicles appearing as white spheres and seemingly a bit unfocused occur upon thick staining.
  d. Some EV should present in a typical cup-like shape.
  e. Best image quality can be obtained in defocus values between −0.8 and −1.5 μM.

## Expected outcomes

This protocol for SEC-based EV isolation approach requires no special laboratory equipment nor specialized laboratory personnel training. The presented EV-analysis methods are based on fundamental biochemical analyses and adhere to current guidelines, providing a framework for multimodal EV quality control. This approach enables assessment of the consistency of EV preparations, which is crucial prior to downstream EV-based assays.

## Limitations

While in-house, manual column preparation and handling is cost effective, it requires careful handling for consistent, reproducible results. Similarly, while gravity-flow SEC is a gentle, non-disruptive EV isolation method, manual fraction collection can lead to differences between operators. Considering that SEC-based EV separation is size-based, non-EV particles such as protein aggregates and lipoproteins might co-isolate with the EVs, necessitating additional steps for the removal of those.

Furthermore, SEC leads to significant dilution of the samples, therefore, depending on the downstream application, samples need to be concentrated (see protocol ‘sample concentration’ above), potentially resulting in a decrease in EV yield. However, the concentration step in parallel can also serve as a mean to remove/decrease the amount of unwanted sample components and/or perform buffer exchange.

## Troubleshooting

### Problem 1

Visible loss of Sepharose from column.

### Potential solution

Insufficient coverage of the syringe exit by the nylon filter can lead to loss of Sepharose from the syringe. To avoid this, make sure to use a filter piece of the right size (Step 2.c), which completely covers the syringe exit but does not curl up on the walls of the syringe. Ensure that the filter lays smoothly on the syringe bottom covering the exit by carefully pipetting approximately 2 ml filtered DPBS on top of the nylon filter (Step 2.e). Take special care when adding the first 2-3 ml of Sepharose not to move and/or lift up the filter.

### Problem 2

Poor SEC separation of EVs.

### Potential solution

When preparing SEC columns ensure that the washed Sepharose equilibrates to working temperature, and the loading of the column is continuous in order to avoid formation of layers/interfaces within the column bed (Step 2.f). Similarly, prior to performing SEC let the column and the wash buffer (DPBS) equilibrate to working temperature to avoid bubble formation within the column matrix (Step 5.). Alternatively, plasma samples can also be equilibrated to working temperature. However, not cooling the samples for extended time might impact sample quality. It is crucial that the plasma samples loaded onto the SEC columns are free from debris and other inconsistencies, therefore it is important to adhere to the centrifugation steps described (Step 4., troubleshooting problem 3.).

### Problem 3

Cloudy plasma samples following second centrifugation step prior to loading on SEC column.

### Potential solution

Make sure not to disturb the pellet following first centrifugation step (Step 4.). Leave a thin layer of liquid on pellet when transferring supernatant to new tube. If necessary, introduce a third centrifugation round with same centrifugation time and speed.

### Problem 4

Problem with detecting EV markers via SDS-PAGE and WB analysis.

### Potential solution

Minimize protein degradation by ensuring adequate sample storage and handling. I.e. aim at further processing of collected fractions into concentrated EV samples without delay (Step 6.), perform centrifugation in cooled (4°C) centrifuge (Steps 20. and 21.), prepare aliquots of concentrated EV samples to avoid multiple freeze-thaw cycles, and store those at −80°C (Step 22.). When handling EV samples store them on ice and avoid extensive vortexing. Make sure to use sample loading-, running-, and transfer-buffer compatible with gel, running- and blotting-system of choice. Prepare samples for loading in accord with recommendations for the used primary antibody (reducing or non-reducing conditions) (Step 15.), and optimize antibody dilution and incubation parameters if necessary.

### Problem 5

Artefacts during NTA measurements.

### Potential solution

Avoid extensive vortexing of the diluted EV samples prior to loading into the instrument, as microbubbles can hinder the readings. Make sure to rinse well the instrument prior to use and in between samples. Make sure to use filtered, particle free buffer (DPBS) for sample dilution and rinsing (Step 25.).

## Resource availability

### Lead contact

Requests for further information and resources should be directed to and will be fulfilled by the lead contact, Friederike Zunke (friederike.zunke@fau.de).

### Technical contact

Questions about the technical specifics of performing the EV SEC, NTA, and WB protocols should be directed to the technical contact, Fanni Annamária Boros (fanniannamaria.boros@uk-erlangen.de). Questions about the technical specifics of performing TEM analysis should be directed to the technical contact, Philipp Arnold (philipp.arnold@fau.de).

### Materials availability

This study did not generate new unique reagents.

### Data and code availability

This study did not generate code or analyzed datasets.

## Acknowledgments

F.A.B. was funded by the 10.13039/501100009379Interdisciplinary Center for Clinical Research (Interdisziplinäre Zentrum für Klinische Forschung; IZKF) at the University Hospital of the University of Erlangen-Nuremberg, through the Junior Research Project J109. Further support was granted through funding by the Michael J. Fox Foundation (MJFF-021325) to P.A. and F.Z., as well as by the 10.13039/501100001659Deutsche Forschungsgemeinschaft (10.13039/501100001659DFG, 10.13039/501100001659German Research Foundation, 505539112, KFO 5024 [A02]) to FZ. M.R. was supported by the Federal Ministry of Research, Technology and Space (ACS_iIMMUNE, 01E02105).

We thank Holger Meixner and Anna Stiegler for their contributions in blood sample collection and processing and Linnea Meinhart for her contribution in testing and validating the isolation protocol. We thank Jan Van Deun for the access to ZetaView instrument and for the help with NTA measurements. Furthermore, we thank all donors of this study. Biorender.com was used in the creation of the graphical abstract.

## Author contributions

F.A.B.: conceptualization, data curation, funding acquisition, investigation and methodology (SEC, SDS-PAGE and WB analysis, NTA), visualization, writing – original draft, and writing – review and editing; P.A.: data curation, investigation, methodology, visualization, writing – original draft (TEM analysis), and writing – review and editing; M.R.: resources and supervision (collection and storage of plasma samples), data curation (updating and maintaining samples and database of Departmental Biobank), and writing – review and editing; F.Z.: conceptualization, funding acquisition, resources, supervision, and writing – review and editing.

## Declaration of interests

The authors declare no competing interests.
